# Supplementary material for: Quantifying alcohol audio-visual content in UK broadcasts of the 2018 Formula 1 Championship: a content analysis and population exposure
Source: BMJ Open. 2020 Aug 7;10(8):e037035. doi: 10.1136/bmjopen-2020-037035 (PMC7418687; doi:10.1136/bmjopen-2020-037035)
Supplement: Supplementary data [file bmjopen-2020-037035supp001.pdf]

Supplementary table 1: Gross and per capita impressions per race by age

	Total						Child <16						Adult >16					
**Race**	**Gross impressions (Million)**	**95% Confidence Interval**		**Per Capita Impressions**	**95% Confidence Interval**		**Gross impressions (Million)**	**95% Confidence Interval**		**Per Capita Impressions**	**95% Confidence Interval**		**Gross impressions (Million)**	**95% Confidence Interval**		**Per Capita Impressions**	**95% Confidence Interval**	
Australia	170.91	153.96	187.87	2.57	2.32	2.83	8.47	6.81	10.14	0.67	0.54	0.80	995.23	962.19	1028.26	18.50	17.88	19.11
Bahrain	61.65	65.86	66.45	0.93	0.86	1.00	3.70	3.17	4.22	0.29	0.25	0.33	353.73	345.15	362.31	6.57	6.42	6.73
China	213.08	192.98	233.19	3.21	2.91	3.51	8.54	6.79	10.32	0.68	0.54	0.82	1264.72	1225.82	1303.63	23.51	22.78	24.23
Azerbaijan	268.28	247.49	289.07	4.04	3.73	4.35	17.55	15.20	19.91	1.39	1.21	1.58	1528.01	1490.98	1565.04	28.40	27.71	29.09
Spain	132.58	121.36	143.81	2.00	1.83	2.17	6.21	5.13	7.28	0.49	0.41	0.58	752.84	732.14	773.54	13.99	13.61	14.38
Monaco	358.17	327.80	388.53	5.39	4.94	5.85	12.29	9.80	14.78	0.98	0.78	1.17	2105.66	2049.07	2162.26	39.14	38.09	40.19
Canada	129.43	113.82	145.03	1.95	1.71	2.18	2.60	1.63	3.58	0.21	0.13	0.28	751.62	720.08	783.15	13.97	13.38	14.56
France	23.50	20.84	26.17	0.35	0.31	0.39	0.51	0.34	0.69	0.04	0.03	0.05	134.46	129.15	139.76	2.50	2.40	2.60
Austria	169.89	153.08	186.69	2.56	2.31	2.81	6.94	5.45	8.44	0.55	0.43	0.67	993.01	960.25	1025.76	18.46	17.85	19.07
Britain	370.60	341.39	399.80	5.58	5.14	6.02	17.42	14.61	20.23	1.38	1.16	1.61	2140.79	2088.19	2193.39	39.79	38.81	40.77
German	185.28	169.60	200.96	2.79	2.55	3.03	5.57	4.36	6.77	0.44	0.35	0.54	1077.65	1048.54	1106.76	20.03	19.49	20.57
Hungarian	139.10	126.54	151.66	2.09	1.91	2.28	5.15	4.08	6.22	0.41	0.32	0.49	788.15	764.51	811.79	14.65	14.21	15.09
Belgium	357.95	328.96	386.94	5.39	4.95	5.83	12.07	9.71	14.44	0.96	0.77	1.15	2143.36	2090.07	2196.66	39.84	38.85	40.83
Italy	253.27	232.28	274.26	3.81	3.50	4.13	10.77	8.85	12.69	0.85	0.70	1.01	1477.27	1438.58	1515.97	27.46	26.74	28.18
Singapore	327.03	298.79	355.28	4.93	4.50	5.35	11.34	9.01	13.67	0.09	0.72	1.09	1886.64	1833.94	1939.34	35.07	34.09	36.05
Russia	93.96	85.08	102.84	1.42	1.28	1.55	4.73	3.86	5.61	0.38	0.31	0.45	535.42	518.47	552.37	9.95	9.64	10.27
Japan	81.51	70.29	92.74	1.23	1.06	1.40	4.84	3.64	6.04	0.38	0.29	0.48	454.81	432.13	477.48	8.45	8.03	8.88
USA	285.63	263.68	307.59	4.30	3.97	4.63	8.48	6.80	10.16	0.67	0.54	0.81	1642.94	1604.88	1683.01	30.56	29.83	31.28
Mexico	113.24	98.16	128.33	1.71	1.48	1.93	2.96	1.89	4.03	0.23	0.15	0.32	651.52	620.74	682.31	12.11	11.54	12.68
Brazil	129.62	112.77	146.47	1.95	1.70	2.21	1.70	0.85	2.55	0.14	0.07	0.20	739.06	704.91	773.21	13.74	13.10	14.37
Abu Dhabi	61.55	56.13	66.97	0.93	0.85	1.01	2.49	2.01	2.97	0.20	0.16	0.24	349.78	339.66	359.90	6.50	6.31	6.69
