# Supplementary material for: Quantifying alcohol audio-visual content in UK broadcasts of the 2018 Formula 1 Championship: a content analysis and population exposure
Source: BMJ Open. 2020 Aug 7;10(8):e037035. doi: 10.1136/bmjopen-2020-037035 (PMC7418687; doi:10.1136/bmjopen-2020-037035)
Supplement: Author's manuscript [file bmjopen-2020-037035.draft_revisions.pdf]

BMJ Open

BMJ Open is committed to open peer review. As part of this commitment we make the peer review history of every article we publish publicly available.

When an article is published we post the peer reviewers' comments and the authors' responses online. We also post the versions of the paper that were used during peer review. These are the versions that the peer review comments apply to.

The versions of the paper that follow are the versions that were submitted during the peer review process. They are not the versions of record or the final published versions. They should not be cited or distributed as the published version of this manuscript.

BMJ Open is an open access journal and the full, final, typeset and author-corrected version of record of the manuscript is available on our site with no access controls, subscription charges or pay-per-view fees (http://bmjopen.bmj.com).

If you have any questions on BMJ Open's open peer review process please email info.bmjopen@bmj.com

BMJ Open

Quantifying, and estimating UK population exposure to, alcohol audio-visual content in UK broadcasts of the 2018 Formula 1 Championship.

Journal:	*BMJ Open*
Manuscript ID	bmjopen-2020-037035
Article Type:	Original research
Date Submitted by the Author:	16-Jan-2020

Complete List of Authors:	Barker, Alexander; University of Nottingham, Division of Epidemiology and Public Health<br>Opazo-Breton, Magdalena; University of Nottingham, Division of Epidemiology and Public Health<br>Thomson, Emily; University of Nottingham<br>Britton, John; University of Nottingham, Division of Epidemiology<br>Grant-Braham, Bruce; Bournemouth University - Talbot Campus<br>Murray, Rachael; University of Nottingham, Division of Epidemiology and Public Health

Keywords:	EPIDEMIOLOGY, PUBLIC HEALTH, STATISTICS & RESEARCH METHODS

SCHOLARONE™
Manuscripts

I, the Submitting Author has the right to grant and does grant on behalf of all authors of the Work (as defined in the below author licence), an exclusive licence and/or a non-exclusive licence for contributions from authors who are: i) UK Crown employees; ii) where BMJ has agreed a CC-BY licence shall apply, and/or iii) in accordance with the terms applicable for US Federal Government officers or employees acting as part of their official duties; on a worldwide, perpetual, irrevocable, royalty-free basis to BMJ Publishing Group Ltd ("BMJ") its licensees and where the relevant Journal is co-owned by BMJ to the co-owners of the Journal, to publish the Work in this journal and any other BMJ products and to exploit all rights, as set out in our licence.

The Submitting Author accepts and understands that any supply made under these terms is made by BMJ to the Submitting Author unless you are acting as an employee on behalf of your employer or a postgraduate student of an affiliated institution which is paying any applicable article publishing charge ("APC") for Open Access articles. Where the Submitting Author wishes to make the Work available on an Open Access basis (and intends to pay the relevant APC), the terms of reuse of such Open Access shall be governed by a Creative Commons licence – details of these licences and which Creative Commons licence will apply to this Work are set out in our licence referred to above.

Other than as permitted in any relevant BMJ Author's Self Archiving Policies, I confirm this Work has not been accepted for publication elsewhere, is not being considered for publication elsewhere and does not duplicate material already published. I confirm all authors consent to publication of this Work and authorise the granting of this licence.

For peer review only

Quantifying and estimating UK population exposure to alcohol audio-visual content in UK broadcasts of the 2018 Formula 1 Championship.

Alexander B. Barker[1], Magdalena Opazo Breton[1], Emily Thomson[1], John Britton[1], Bruce Grant-Braham[2], Rachael L. Murray[1]

[1]UK Centre for Tobacco and Alcohol Studies, Division of Epidemiology and Public Health, University of Nottingham, Clinical Sciences Building, City Hospital, Nottingham, NG5 1PB, United Kingdom

[2]University of Bournemouth, Dorset House, Talbot Campus, Fern Barrow, Poole, BH12 5BB, United Kingdom

Corresponding Author: Alexander B. Barker, alexander.barker@nottingham.ac.uk

Magdalena Opazo Breton, magdalena.opazo@nottingham.ac.uk

Emily Thomson, mzyet3@exmail.nottingham.ac.uk

John Britton, j.britton@outlook.com

Bruce Grant-Braham, BBraham@bournemouth.ac.uk

Rachael L. Murray, rachael.murray@nottingham.ac.uk

Word Count: 4084(excl. abstract and references)

Declaration of conflict of interests: Nothing to declare

Funding

This work was supported by the Medical Research Council [grant number MR/K023195/1] and the UK Centre for Tobacco and Alcohol Studies, with core funding from the British Heart Foundation, Cancer Research UK, Economic and Social Research Council and the Department of Health under the auspices of the UK Clinical Research Collaboration. The funders had no role in the study design, data collection and analysis, decision to publish or preparation of the manuscript.

For peer review only - http://bmjopen.bmj.com/site/about/guidelines.xhtml

Abstract

Objectives: Exposure to alcohol imagery is associated with subsequent alcohol use among young people, and UK broadcasting regulations protect young people from advertising and alcohol content in UK television. However, alcohol promotion during sporting events, a significant potential medium of advertising to children, is exempt. We report an analysis of and estimate the UK population exposure to, alcohol content, including branding, in UK broadcasts of the 2018 F1 Championship.

Setting: UK

Participants: None. Content analysis of broadcast footage of 21 2018 F1 Championship on Channel 4, using 1-minute interval coding of any alcohol content, actual or implied use, other related content or branding. Census data and viewing figures were used to estimate gross and per capita alcohol impressions.

Results: Alcohol content occurred in all races, in 1613 (56%) one-minute intervals of race footage and 44 (13%) of intervals across 28% of advertisement breaks. The most prominent content was branding, occurring in 51% of race intervals and 7% of advertisement break intervals, appearing predominantly on billboard advertisements around the track, with the *Heineken* and *Johnnie Walker* brands being particularly prominent. The 21 races delivered an estimated 3.47 billion alcohol gross impressions (95% CI 3.1 – 3.8) to the UK population, including 136 million (95% CI 109 - 163) to children; and 3.2 billion alcohol gross impressions of alcohol branding, including 125 million impressions to children. Branding was also shown in race footage from countries where alcohol promotion is prohibited.

For peer review only

Conclusions: Alcohol content was highly prevalent in the 2018 F1 Championship broadcasts, delivering millions of alcohol impressions to young viewers. This exposure is likely to represent a significant driver of alcohol consumption among young people.

Article Summary

Strengths and limitations of the study

- This study builds on previous studies by estimating population exposure to alcohol content shown in F1 races
- Established methods were used to explore alcohol content in the 2018 F1 championship races
- This study only explored broadcasts where races occurred and not supplementary programmes
- Future studies should explore sales data to examine whether this exposure led to an increase in sales.

Introduction

Alcohol consumption in the UK is the 8th highest in Europe (1), and currently causes at least 7,000 UK deaths (2) and an estimated economic burden of between £21 and £52 billion each year (3). Preventing alcohol morbidity and mortality is therefore a clear public health priority.

There is strong evidence that exposure to advertising or other alcohol audio visual content (AVC) in the media increases subsequent alcohol use by adolescents (4-7), and both commercial advertising of alcohol and alcohol content in broadcast programmes is regulated in the UK to protect adolescents from exposure. However, Advertising Standards Authority commercial advertising regulatory codes do not cover broadcast footage of imagery arising from sponsorship of sporting events (8, 9); since this is programme content, it is regulated instead by the Office of Communications (Ofcom). The Ofcom Broadcasting

For peer review only - http://bmjopen.bmj.com/site/about/guidelines.xhtml

Code (10) restricts depictions of alcohol misuse in programmes made for children, and the glamorisation of alcohol use in programmes broadcast before the 9 p.m. watershed (11) otherwise likely to be widely seen, heard or accessed by children without editorial justification (Section 1.10). The Code also restricts advertising within programmes, stating that 'no undue prominence may be given in programming to a product, service or trade mark' through the presence of or reference to a product where the is no editorial justification or the manner in which a product, service or trademark appears in programming (Section 9.5). However, Ofcom has no remit over sports sponsorship deals, such as a company sponsoring a stadium, a team or an individual sportsperson, and OfCom guidance states that the context of advertising is taken into account with a greater number of in situ advertising expected at sporting venues (12, 13). Alcohol advertising during televised sporting events is thus a potentially unregulated source of exposure to alcohol content for young people.

Whilst previous research has shown that alcohol content and branding is highly prevalent during F1 Championship races (14), the extent of this exposure to young people remains unknown. We have therefore quantified alcohol AVC in all 21 races from the 2018 F1 Championship and estimated UK population exposure, including child exposure, to this content. We have also compared the amount of alcohol imagery in the 2018 race series with our earlier analysis of footage from the 2017 competition.

Methods

The 2018 Formula 1 Championship took place between 15th March 2018 and 1st December 2018 and featured races in twenty-one countries. Ten of these races were broadcast in the UK live and in full by Channel 4, and eleven in selected highlight form. These broadcasts were recorded, viewed and coded using the one-minute interval period method previously described (14-18), coding the presence of audio-visual alcohol content every one-minute interval in the following categories:

Actual Use: Use of alcohol onscreen by any person (for example, seeing a person actually consume alcohol on screen

Implied Use: Any implied alcohol use without any actual use on screen (for example, seeing a person holding a drink/bottle of alcohol, but not actually consuming alcohol).

Other Alcohol Reference: The presence onscreen of alcohol or other related materials (for example bottles or beer pumps not currently in use, or advertising materials)

Brand Appearance: The presence of clear and unambiguous branding (for example, when a brand is identified on screen). Brand appearances were divided into the following categories; Alcoholic and 0% or low alcohol products, products which, while not an alcohol product, share branding with alcoholic products.

Any Alcohol Content: Any of the above.

For coding purposes, multiple instances of the same category in the same one-minute period were considered to be single event, whilst instances that ran into consecutive one-minute periods were coded as separate events. Instances in different categories in the same interval were recorded as different events. Alcohol content was recorded in two categories in the same interval if the content met the criteria for two categories, for example if a driver

For peer review only

For peer review only - http://bmjopen.bmj.com/site/about/guidelines.xhtml

drank from a branded bottle, this would be recorded as actual use and a brand appearance. Ten percent of the coding was checked by a second author (ET) to ensure accuracy and reliability in the coding method.

We estimated UK audience exposure using viewing data from *Digital.I* (19) and UK mid-year population estimates for 2017 (20) combined with numbers of alcohol appearances to estimate gross and per capita one-minute impressions by age group, using previously reported methods (21-23). The method involves combining viewership (obtained from viewing figures) with the number of intervals containing alcohol appearances per episode to calculate gross impressions as the estimated number of exposures delivered. Dividing gross impressions by population mid-year estimates provided per capita impressions, the estimated number of alcohol impressions delivered to each person. Both gross and per capita impressions were computed by age group. Analyses were conducted in IBM SPSS Statistics (V.24) and Microsoft Excel (2013). The confidence level was set to 95%. We compared content with findings of our earlier studies alcohol content in a sample of F1 races from 2017 (14) using chi-square analysis.

Results

We coded a total of 3396 one-minute intervals, of which 2899 were from race footage, and 497 from advertisement breaks, all of which were broadcast by Channel 4 on a Sunday, with start times ranging from 06:00 to 23:00 and a mean broadcast duration (including advertisement breaks) of 2 hours 21 minutes (range = 1 hour 34 minutes to 3 hours 7 minutes).

For peer review only - http://bmjopen.bmj.com/site/about/guidelines.xhtml

Any Alcohol Content

In total, 1613 (56%) one-minute intervals of racing, and 44 (13%) one-minute advertisement intervals contained any alcohol content, with occurrences in all twenty-one races and 28% of advertisement breaks (Table 1). The majority of alcohol content occurred during race footage.

Table 1 here

Actual Use

Alcohol consumption during race footage was limited to consumption of a sparkling drink on the winner's podium, identified from branding on the bottle or podium as Carbon Champagne, which occurred at the end of eighteen races (86%) in a total of 25 (1%) one-minute intervals. There were ten advertisement breaks depicting alcohol consumption, across 11 one-minute intervals (<1%).

Implied Use

Implied alcohol use was observed in 92 (3%) race intervals, predominantly the sparkling drink being sprayed from the winner's podium. Implied use was seen in 34 one-minute intervals during advertisement breaks, and predominantly comprised imagery of people holding drinks during advertisements for alcohol brands.

Other Alcohol References

Alcohol references, predominantly involving billboard and car brand advertising, occurred in 1571 race intervals (46% of all race intervals), including 'When you drink, never drive' billboards, which appeared in 241 (7%) of race intervals. Alcohol references also occurred in

For peer review only - http://bmjopen.bmj.com/site/about/guidelines.xhtml

38 (8%) one-minute intervals from advertisement breaks, mostly consisting of bottles or branded items (such as beer pumps) (Table 1).

Brand Appearances

Alcohol product branding in billboard advertisements or on the side of cars or racing suits, occurred in 1490 (51%) race intervals across all 21 races. The most common brands observed were *Heineken,* which occurred in 1031 intervals and appeared 8795 times; and *Johnnie Walker,* which appeared in 608 intervals and appeared on screen 4364 times. *Heineken 0.0,* a non-alcoholic lager which shared the *Heineken* brand name and imagery, occurred in 120 one-minute intervals (8% of the 1490 intervals containing branding) and appeared 548 times. Alcohol branding occurred in 37 (7%) advertisement break intervals, with the most common brand being *Heineken* (Table 1). A list of all the brands observed is presented in Figure 1.

Figure 1 here

In addition to billboard advertising, computer-generated advertising added to highlights programmes were seen in 17 intervals during two races (Canada and Austria). These intervals featured a red star, reminiscent of the *Heineken* logo, superimposed over the track (See Figure 2).

Figure 2 here

Differences between race venues

The amount of alcohol content and alcohol branding was highest in Monaco GP and lowest in the Bahrain GP (table 2)

Table 2 here

UK Population Exposure

We estimate that the 21 race broadcasts delivered 3.47 billion alcohol gross impressions (95% CI 3.1 – 3.8) to the UK population, including 136 million (95% CI 109 – 163) to children aged under 16. Alcohol impressions per capita were highest (average 513.11 (95%CI 499.96 – 526.25)) in the 55-64 age group and were higher in men than women (433.50 (95% CI 420.98 – 446.02) and (206.52 (95%CI 197.05 – 215.99) respectively). Children received an average 12.25 (95% CI 9.84 – 14.66) per capita impressions. Of these impressions, 3.2 billion (95% CI 2.9 – 3.5) were of alcohol branding and including 125 million (95% CI 100 – 149) to children. (See supplementary table 1 for a breakdown of gross and per capita impressions per race). We estimate that the computer-generated branding features in 2 races delivered 24 million gross impressions to the UK population including 664 thousand to children.

Comparison to 2017 Championship

Comparison with our earlier analysis of alcohol content in the 2017 F1 championship (14) demonstrated significantly greater alcohol content in the 2018 season, particularly in relation to the proportion of intervals including alcohol branding, and especially Heineken advertising (Table 3). Heineken 0.0 advertising was significantly less prevalent in the 2018 footage (Table 3). In our previous content analysis (14), Heineken 0.0 branding was seen at only 2 events, the Spanish Grand Prix and the Monaco Grand Prix. In the 2018 Monaco Grand Prix, billboard advertising for Heineken 0.0 had been replaced with conventional Heineken imagery (See figure 3).

Table 3 here

Figure 3 here

Discussion

This study demonstrates that audio-visual alcohol content, including branding and particularly *Heineken* and *Johnnie Walker* branding, was highly prevalent in broadcast footage of the 2018 F1 championship races, and that the overall amount of alcohol content

For peer review only - http://bmjopen.bmj.com/site/about/guidelines.xhtml

in 2018 was greater than in 2017 (14), largely because of substitution of zero-alcohol with alcoholic *Heineken* branding. The alcohol content in the broadcast race footage generates substantial population exposure, delivering billions of viewer impressions. These included more than 100 million gross branded alcohol impressions to children aged under 16.

Our study thus provides evidence that UK broadcast footage of the F1 Championship is a significant source of exposure to alcohol content and advertising for children. Alcohol content was prevalent in all races, even races which took place in countries with alcohol advertising restrictions. Alcohol advertising during these events appears to be changing with the inclusion of computer-generated alcohol advertising, which was introduced in the 2018 Championship. Since the F1 Championships are international events with global audiences, the UK population exposure figures likely represent a very small proportion of the true total global exposure.

Some alcohol producers associate their advertising with responsible drinking messages. Heineken's 5-year F1 sponsorship has been heavily criticised *for "*linking a popular motor sport to a significant cause of avoidable physical, mental and social harm and more specifically one of the major killers on our roads, drink driving"(24). This global campaign incorporates Heineken's characteristic red star and green branding on billboards which also have a prominent *"When You Drive, Never Drink"* message (25). Indeed Heineken has said that it will use F1 to promote this campaign (26) and the *Heineken 0.0* brand (27). We have previously argued that *Heineken 0.0* advertising may be acting as an alibi for alcohol products (14), not least because they share the same brand imagery (a star in the case of Heineken) on both alcohol and low alcohol products, potential consumers, and particularly young people, may well not distinguish advertising for alcoholic and non-

For peer review only - http://bmjopen.bmj.com/site/about/guidelines.xhtml

alcoholic *Heineken* products. This industry tactic could potentially be serving a subtle public relations function, engendering goodwill with potential consumers of the brand while increasing their promotion of their alcohol products, promoting brand preference and product consumption (28, 29). The current study has identified that the proportion of *Heineken 0.0* advertising has decreased significantly since our 2017, with billboards for *Heineken 0.0* from 2017 being replaced with regular *Heineken* billboards.

The majority of the F1 Championship races (18 of 21) were broadcast on a Sunday before the 9 p.m. watershed, a time when children are likely to be watching what their parents are watching (30). Channel 4 state that they '*abide*' by the Office of Communications (Ofcom) Broadcasting Code (10), which considers factors that determine whether a programme should be shown including *'the likely number and age range of children watching, taking into account school time, weekends and holidays'(31)*. The current study has demonstrated that the broadcast footage of the F1 Championship is widely seen by children and delivers millions of branded alcohol content impressions to children Furthermore, the Ofcom code also states that *'Before the watershed, …the misuse of alcohol must never be condoned, encouraged or glamorised and scenes showing such material should generally be avoided unless there is editorial justification'* (10). All of the F1 races featured scenes of drivers celebrating their win by drinking from and spraying bottles of champagne, a traditional celebration in F1 (32), which could potentially be associating driving with alcohol use (33) and glamourising alcohol use by showing drivers consuming alcohol in what F1's CEO considers to be a glamourous sport (34).

Rule 9.5 of the Ofcom Broadcasting Code (10) states that 'no undue prominence may be given in programming to a product, service or trade mark' through the presence of or

For peer review only - http://bmjopen.bmj.com/site/about/guidelines.xhtml

reference to a product where the is no editorial justification or the manner in which a product, service or trademark appears in programming. Whilst this rule takes into account the context and in-situ advertising at venues is editorially justified, the current study found that computer generated Heineken branding was added to F1 race highlights programmes, through the inclusion of a red star superimposed over the track similar to Heineken's red star logo (see figure 2) representing an apparent clear breach of the Ofcom broadcasting code. Channel 4 was recently found to be in breach of the code for their Formula 1. We reported this to Ofcom (13) which noted that the two Grands Prix containing computer-generated alcohol advertising were broadcast before an Ofcom ruling which found Channel 4 to be in breach of the code for their F1 coverage featuring computer-generated 'Rolex' branding (a 'Rolex' clock face superimposed over footage of The Singapore Flyer, a large Ferris wheel) (35), and expect broadcasters to take the precedent of the 'Rolex' ruling into account for subsequent broadcasts.

Alcohol content and brand prevalence differed between race venues, possibly due to differences in national restrictions on alcohol advertising (36). The races with the least alcohol content were Bahrain, Abu Dhabi and France; Bahrain and Abu Dhabi are events in Islamic countries were alcohol advertising is prohibited, while France has statutory legislation, 'the Loi Evin'(37), prohibiting alcohol marketing and advertising by placing a total ban on the direct or indirect advertising of all alcoholic beverages over 1.2% ABV on television and prohibiting sponsorship of sports events by alcohol companies (38). Alcohol advertising did occur in UK broadcast footage of races in countries where alcohol advertising is prohibited, such as Russia, France, and Bahrain, raising concerns that alcohol brands are bypassing country-specific regulations in the F1 races. Race footage featuring billboard

For peer review only

advertisements are also broadcast worldwide, which could circumvent alcohol advertising regulations in a similar way to the UK's lack of regulation covering advertisements at sporting venues (8, 9, 12, 13).

Whilst the current study found that alcohol branding was highly prevalent in broadcast footage of the 2018 F1 championship races, we currently do not know whether this led to increased alcohol sales. Future studies should explore sales data for prevalent brands to identify if the presence of branding in such events led to an increase in sales of branded goods featured in the Championship races. Channel 4 also broadcast other F1 programmes, including qualifying race and pre and post-event programmes, in this content analysis we focused on the broadcast containing the actual race as specified by Channel 4, rather than qualifying or post event programmes. It is therefore likely that this content analysis underestimates the amount of alcohol AVC shown in the F1 Championship as a whole, and the population exposure to this content.

Conclusion

Alcohol content shown on TV, including alcohol advertisements and branding, is known to have an effect on the uptake of alcohol use in young people (39). Alcohol content broadcast during the F1 races resulted in millions of gross alcohol content impressions being delivered to children, and this exposure is likely to lead to subsequent alcohol use. We also found computer-generated branding in the Formula 1 races broadcast in the UK, contravening an earlier Ofcom ruling, and alcohol advertising in countries where this is prohibited.

For peer review only - http://bmjopen.bmj.com/site/about/guidelines.xhtml

Restrictions on and enforcement of alcohol AVC during sporting events are needed to protect children and adolescents from this avenue of alcohol advertising.

Declarations

Patient consent for publication: Not required.

Competing interests: The authors declare that they have no competing interests.

Funding: This work was supported by the Medical Research Council [grant number MR/K023195/1] and the UK Centre for Tobacco and Alcohol Studies, with core funding from the British Heart Foundation, Cancer Research UK, Economic and Social Research Council and the Department of Health under the auspices of the UK Clinical Research Collaboration. The funders had no role in the study design, data collection and analysis, decision to publish or preparation of the manuscript.

Author's contributions: ABB led the data coding and data analysis and contributed to the manuscript. ET double coded the data to ensure reliability. MOP supported data analysis. BG contributed with interpreting the findings of the study. JB and RM contributed to drafting the manuscript. All authors read and approved the final manuscript.

Acknowledgements: Not applicable

Availability of data and materials: Data and materials are available from the lead author upon reasonable request.

For peer review only - http://bmjopen.bmj.com/site/about/guidelines.xhtml

References

1. United European Gastroenterology. Alcohol and Digestive Cancers 2017 [Available from: https://www.ueg.eu/publications/alcohol-and-digestive-cancers-report/.
2. Office on National Statistics. Statistics on Alcohol; England 2017. http://www.content.digital.nhs.uk/catalogue/PUB23940/alc-eng-2017-rep.pdf: NHS England; 2017.
3. Public Health England. The public health burden of alcohol and the effectiveness and cost-effectiveness of alcohol control policies: an evidence review. 2016.
4. Anderson P, De Brujin A, Angus K, Gordon R, Hastings G. Impact of alcohol advertising and media exposure on adolescent alcohol use: a systematic review of longitudinal studies. Alcohol and Alcoholism. 2009;44(3):229-43.
5. Smith L, Foxcroft DR. The effects of alcohol advertising, marketing and portrayal on drinkin behaviour in young people: systematic review of prospective cohort studies. BMC Public Health. 2009;9(51):1-11.
6. Hanewinkel R, Sargent JD, Hunt K, Sweeting H, Engels RC, Scholte RH. Portrayal of alcohol consumption in movies and drinking initiation in low-risk adolescents. Pediatrics. 2014;133:973-82.
7. Chang F, Miao N, Lee C, Chiu C, Lee S. The association of media exposure and media literacy with adolescent alcohol and tobacco use. Journal of Health Psychology. 2016;21(4):513-25.
8. Advertising Standards Authority. The CAP code 2019 [Available from: https://www.asa.org.uk/type/non_broadcast/code_folder/scope-of-the-code.html.
9. Advertising Standards Authority. The Broadcast Code 2019 [Available from: https://www.asa.org.uk/codes-and-rulings/advertising-codes/broadcast-code.html.
10. Ofcom. The Ofcom Broadcasting Code (with the Cross-promotion Code and the On Demand Programme Service Rules) 2017 [Available from: https://www.ofcom.org.uk/tv-radio-and-on-demand/broadcast-codes/broadcast-code.
11. Ofcom. What is the Watershed? 2013 [Available from: https://www.ofcom.org.uk/tv-radio-and-on-demand/advice-for-consumers/television/what-is-the-watershed.
12. Ofcom. Guidance Notes: Section Nine: Commercial references in television programming 2016 [Available from: https://www.ofcom.org.uk/__data/assets/pdf_file/0014/33611/section9_may16.pdf.
13. Ingram P. Letter regarding Heineken branding in Channel four's coverage of Formula 1, Ref.: 00626184 2018 [Available from: https://ukctas.net/pdfs/20181115-ofcom-letter-to-Alex-Barker.pdf.
14. Barker AB, Britton J, Grant-Braham B, Murray RL. Alcohol Audio-Visual Content in Formula 1 television broadcasting. BMC Public Health. 2018;18(1155).
15. Lyons A, McNeill A, Britton J. Tobacco imagery on prime time UK television. Tobacco Control. 2013;23(3):257-63.
16. Barker AB, Whittamore KH, Britton J, Murray RL, Cranwell J. Audio-visual alcohol content in UK television. Journal of Public Health. 2018;Online First.
17. Barker AB, Smith J, Hunter A, Britton J, Murray RL. Tobacco and alcohol audio-visual content in Netflix and Amazon Prime Instant Video original programming. BMJ Open. 2019.
18. Barker AB, Whittamore KH, Britton J, Cranwell J. A content analysis of tobacco content in UK television. Tobacco Control. 2018;Online First.
19. Digital I. Digital.I: A fresh perspective on TV viewing figures 2018 [Available from: http://www.digital-i.com.
20. Office for National Statistics. Population estimates for the UK, England and Wales, Scotland and Northern Ireland: Mid 2017 2018 [Available from: https://www.ons.gov.uk/peoplepopulationandcommunity/populationandmigration/populationestimates/bulletins/annualmidyearpopulationestimates/mid2017.
21. Cranwell J, Opazo-Breton M, Britton J. Adult and adolescent exposure to tobacco and alcohol content in contemporary YouTube music videos in Great Britain: a population estimate. J Epidemiol Community Health. 2016;70(5):488-92.

For peer review only - http://bmjopen.bmj.com/site/about/guidelines.xhtml

22. Barker AB, Opazo Breton M, Cranwell J, Britton J, Murray RL. Population exposure to smoking and tobacco branding in the UK reality show 'Love Island'. Tobacco Control. 2018.
23. Sargent JD, Tanski SE, Gibson J. Exposure to movie smoking among US adolescents aged 10 to 14 years: a population estimate. Pediatrics. 2007;119(5):e1167-76.
24. Crash. Heineken F1 deal leads to fresh calls for alcohol sponsorship ban 2016 [Available from: https://www.crash.net/f1/news/231262/1/heineken-f1-deal-leads-to-fresh-calls-for-alcohol-sponsorship-ban.
25. Eurocare. Formula 1 puts Heineken in the driving seat 2016 [Available from: http://www.eurocare.org/media_centre/press_releases/formula_1_puts_heineken_in_the_driving_seat.
26. Heineken. When You Drive, Never Drink. 2015;2018.
27. Heineken. Heineken 0.0: Great taste, all natural, only 69 calories and zero alcohol 2017 [Available from: https://www.theheinekencompany.com/media/media-releases/press-releases/2017/05/2104120.
28. Smith SW, Atkin CK, Roznowski J. Are 'Drink Responsibly' alcohol campaigns strategically ambiguous? Health Communication. 2006;20(1):1-11.
29. Jones SC, Hall S, Kypri K. Should I drink responsibly, safely or properly? Confusing messages about reducing alcohol related harm. PLOS One. 2017;12(9):e0184705.
30. Jago R, Thompson J, Sebire S, Wood L, Pool L, Zahra J, et al. Cross-sectional associations between the screen-time of parents and young children: differences by parent and child gender and day of the week. International Journal of Behavioral Nutrition and Physical Activity. 2014;11(54):1-8.
31. Channel 4. Producers Handbook 2017 [Available from: http://www.channel4.com/producers-handbook/.
32. Times T. Obituary: Dan Gurney 2018 [Available from: https://www.thetimes.co.uk/article/obituary-dan-gurney-c689k76k6.
33. Eurocare. Alcohol advertising and sponsorship in Formula One: A Dangerous Cocktail 2015 [Available from: http://www.ias.org.uk/uploads/pdf/IAS%20reports/ALCOHOL_ADVERTISING_AND_SPONSORSHIP_IN_FORMULA_ONE__A_DANGEROUS_COCKTAIL_MAY_2015.pdf.
34. Tass: Russian News Agency. Formula One racing to remain 'glamorous sport' even without grid girls, vows F1 CEO Carey [Available from: https://tass.com/sport/989870.
35. Ofcom. Ofcom broadcast and on demand bulletin 2018 [Page 41]. Available from: https://www.ofcom.org.uk/__data/assets/pdf_file/0027/117468/broadcast-on-demand-bulletin-issue-359.pdf.
36. World Health Organisation. Management of substance abuse: country profiles 2014 2014 [Available from: http://www.who.int/substance_abuse/publications/global_alcohol_report/profiles/en/.
37. Court of Justice of the European Communities: Opinion of First Advocate General in Cases C-262/02 and C-429/02, (2004).
38. Institute of Alcohol Studies. Alcohol advertising in the European Union 2019 [Available from: http://www.ias.org.uk/Alcohol-knowledge-centre/Marketing/Factsheets/Alcohol-advertising-in-the-European-Union.aspx.
39. Morgenstern M, Isansee B, Sargent JD, Hanewinkel R. Exposure to alcohol advertising and teen drinking. Preventive Medicine. 2011;2(1):146-51.

For peer review only - http://bmjopen.bmj.com/site/about/guidelines.xhtml

Table 1: Alcohol AVC content by type and location

Type of content	During race broadcast<br><br>N (%)	During advertisements<br><br>N (%)
Any Alcohol Content	1613 (56)	44 (9)
Actual Use	27 (1)	2 (2)
Implied use	92 (3)	34 (7)
Other Alcohol References	1571 (54)	38 (8)
Brand Appearance	1490 (51)	37 (7)
Heineken branding	1029 (35)	2 (2)

For peer review only

Table 2: Comparison of alcohol content between race venues (in order of broadcast)

	Number of 1-minute intervals (% proportion of race intervals)																				
	**Australia**	**Bahrain**	**China**	**Azerbaijan**	**Spain**	**Monaco**	**Canada**	**France**	**Austria**	**Britain**	**Germany**	**Hungary**	**Belgium**	**Italy**	**Singapore**	**Russia**	**Japan**	**USA**	**Mexico**	**Brazil**	**Abu Dhabi**
Any Alcohol Content	79 (55%)	18 (12%)	92 (73%)	81 (43%)	48 (45%)	129 (80%)	90 (96%)	15 (15%)	82 (49%)	110 (66%)	65 (55%)	55 (48%)	117 (74%)	88 (76%)	122 (71%)	40 (34%)	75 (50%)	84 (55%)	97 (83%)	102 (87%)	24 (15%)
Alcohol Use	1 (1%)	0 (0%)	1 (1%)	1 (1%)	2 (2%)	3 (2%)	1 (1%)	2 (2%)	2 (1%)	1 (1%)	1 (1%)	1 (1%)	2 (1%)	1 (1%)	1 (1%)	2 (2%)	1 (1%)	1 (1%)	1 (1%)	1 (1%)	1 (1%)
Implied Use	5 (1%)	1 (1%)	2 (2%)	4 (2%)	7 (7%)	5 (3%)	5 (5%)	4 (4%)	2 (1%)	2 (1%)	4 (3%)	5 (4%)	2 (1%)	6 (5%)	0 (0%)	5 (4%)	1 (1%)	5 (3%)	13 (11%)	10 (9%)	4 (2%)
Other Alcohol References	76 (53%)	13 (9%)	92 (73%)	77 (41%)	43 (41%)	126 (78%)	90 (96%)	15 (15%)	80 (48%)	109 (66%)	63 (53%)	53 (46%)	117 (74%)	87 (75%)	121 (70%)	38 (33%)	75 (50%)	82 (54%)	91 (78%)	102 (87%)	21 (13%)
Brand Appearance	59 (41%)	10 (7%)	92 (73%)	68 (36%)	39 (37%)	121 (75%)	84 (89%)	11 (11%)	79 (47%)	108 (66%)	62 (53%)	48 (42%)	114 (72%)	87 (75%)	121 (70%)	36 (31%)	63 (42%)	82 (54%)	89 (76%)	101 (86%)	16 (10%)
Any Alcohol Content	79 (55%)	18 (12%)	92 (73%)	81 (43%)	48 (45%)	129 (80%)	90 (96%)	15 (15%)	82 (49%)	110 (66%)	65 (55%)	55 (48%)	117 (74%)	88 (76%)	122 (71%)	40 (34%)	75 (50%)	84 (55%)	97 (83%)	102 (87%)	24 (15%)
Alcohol Use	1 (1%)	0 (0%)	1 (1%)	1 (1%)	2 (2%)	3 (2%)	1 (1%)	2 (2%)	2 (1%)	1 (1%)	1 (1%)	1 (1%)	2 (1%)	1 (1%)	1 (1%)	2 (2%)	1 (1%)	1 (1%)	1 (1%)	1 (1%)	1 (1%)
Implied Use	5 (1%)	1 (1%)	2 (2%)	4 (2%)	7 (7%)	5 (3%)	5 (5%)	4 (4%)	2 (1%)	2 (1%)	4 (3%)	5 (4%)	2 (1%)	6 (5%)	0 (0%)	5 (4%)	1 (1%)	5 (3%)	13 (11%)	10 (9%)	4 (2%)
Other Alcohol References	76 (53%)	13 (9%)	92 (73%)	77 (41%)	43 (41%)	126 (78%)	90 (96%)	15 (15%)	80 (48%)	109 (66%)	63 (53%)	53 (46%)	117 (74%)	87 (75%)	121 (70%)	38 (33%)	75 (50%)	82 (54%)	91 (78%)	102 (87%)	21 (13%)
Brand Appearance	59 (41%)	10 (7%)	92 (73%)	68 (36%)	39 (37%)	121 (75%)	84 (89%)	11 (11%)	79 (47%)	108 (66%)	62 (53%)	48 (42%)	114 (72%)	87 (75%)	121 (70%)	36 (31%)	63 (42%)	82 (54%)	89 (76%)	101 (86%)	16 (10%)
Heineken	33 (23%)	0 (0%)	85 (67%)	59 (32%)	0 (0%)	114 (70%)	82 (87%)	1 (1%)	69 (41%)	82 (49%)	14 (12%)	40 (35%)	44 (28%)	74 (64%)	86 (50%)	1 (1%)	65 (43%)	12 (8%)	71 (60%)	96 (82%)	1 (1%)
Heineken 0.0	0 (0%)	0 (0%)	0 (0%)	0 (0%)	30 (28%)	0 (0%)	0 (0%)	0 (0%)	0 (0%)	0 (0%)	0 (0%)	0 (0%)	0 (0%)	36 (31%)	0 (0%)	31 (26%)	0 (0%)	0 (0%)	0 (0%)	0 (0%)	0 (0%)

For peer review only

Table 3: Proportion (%) of race footage containing alcohol content in the 2018 and 2017 Championships.

	2018 Championship	2017 Championship	p-value
**Alcohol**			
Any Alcohol Content	56%	46%	<0.01
Actual Alcohol Use	1%	1%	1
Implied Alcohol Use	3%	4%	0.1498
Other Alcohol reference	54%	44%	<0.01
Alcohol Branding	51%	35%	<0.01
Heineken	35%	13%	<0.01
Heineken 0.0	3%	14%	<0.01

For peer review only

Figure 1: Brands identified in race footage and advertisements

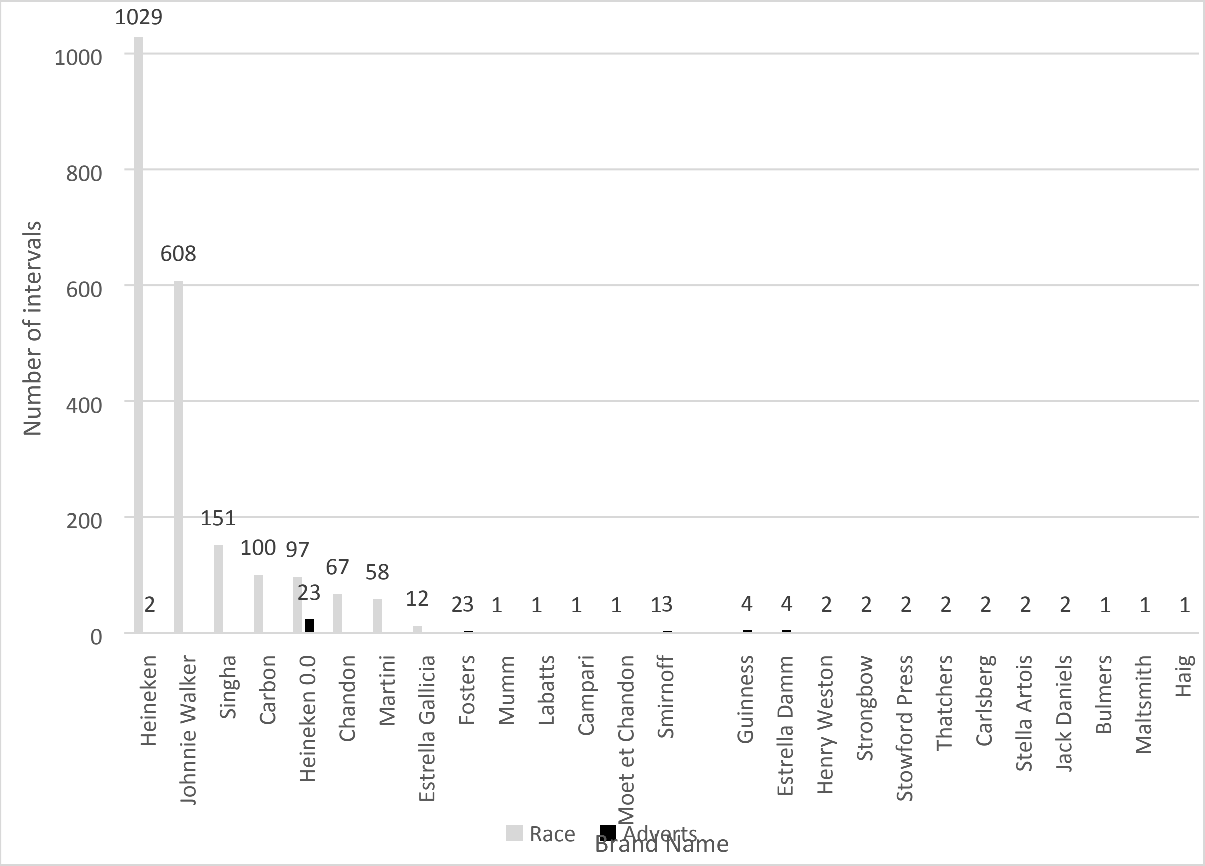

Figure 2: Examples of computer-generated alcohol advertising

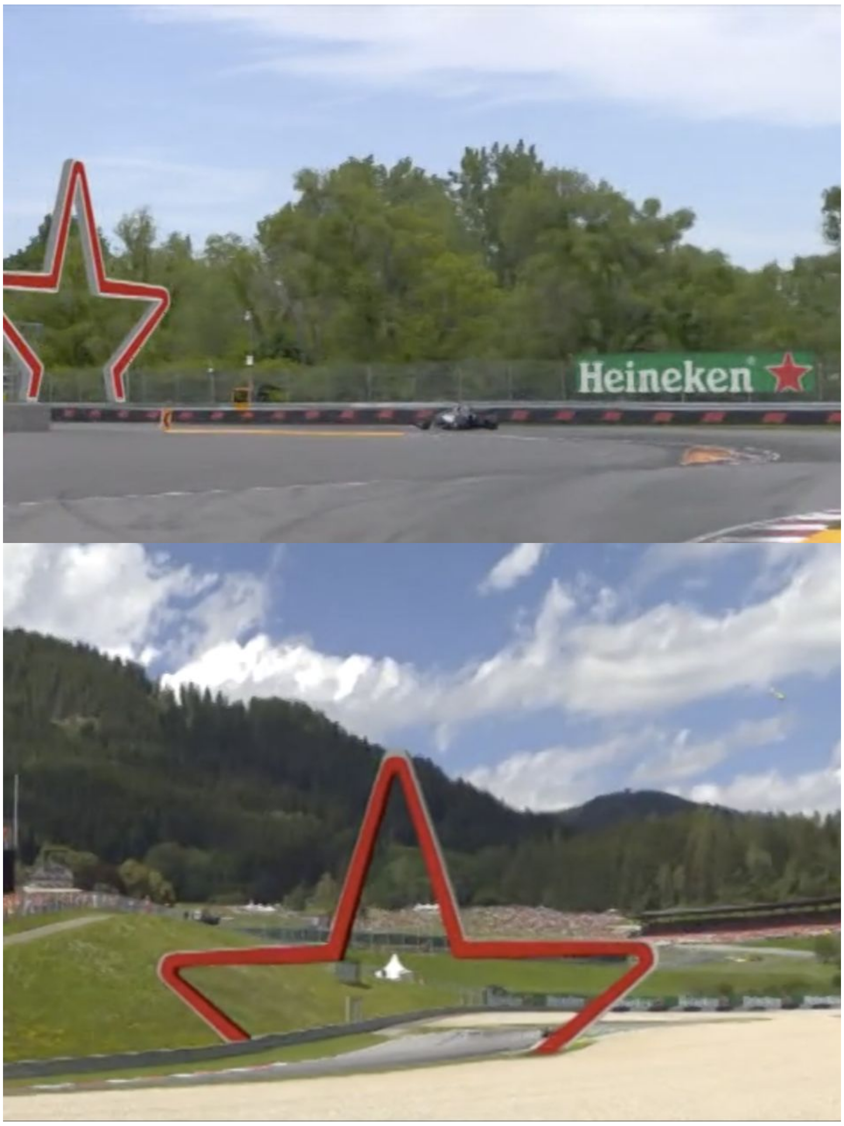

Figure 3: Comparison between Heineken advertising in 2017 compared to 2018 (Both images taken at the same section of track)

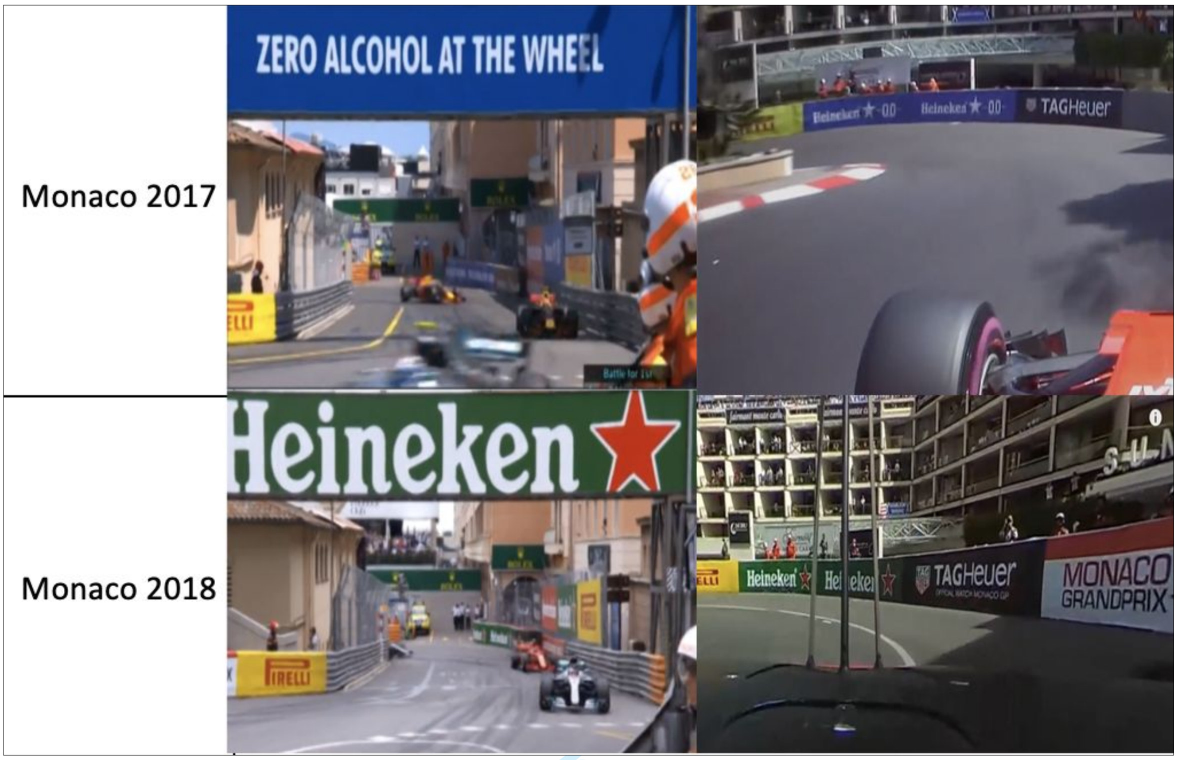

For peer review only - http://bmjopen.bmj.com/site/about/guidelines.xhtml

Supplementary table 1: Gross and per capita impressions per race by age

	Total						Child <16						Adult >16					
**Race**	**Gross impressions (Million)**	**95% Confidence Interval**		**Per Capita Impressions**	**95% Confidence Interval**		**Gross impressions (Million)**	**95% Confidence Interval**		**Per Capita Impressions**	**95% Confidence Interval**		**Gross impressions (Million)**	**95% Confidence Interval**		**Per Capita Impressions**	**95% Confidence Interval**	
Australia	151.09	136.10	166.09	2.57	2.32	2.83	7.47	6.00	8.93	0.67	0.54	0.80	878.69	849.52	907.85	18.50	17.88	19.11
Bahrain	54.50	50.26	58.74	0.93	0.86	1.00	3.26	2.80	3.71	0.29	0.25	0.33	312.31	304.74	319.88	6.57	6.42	6.73
China	188.37	170.60	206.15	3.21	2.91	3.51	7.52	5.96	9.09	0.68	0.54	0.82	1116.62	1082.27	1150.97	23.51	22.78	24.23
Azerbaijan	237.17	218.79	255.55	4.04	3.73	4.35	15.46	13.39	17.54	1.39	1.21	1.58	1349.08	1316.39	1381.77	28.40	27.71	29.09
Spain	117.21	107.29	127.13	2.00	1.83	2.17	5.47	4.52	6.41	0.49	0.41	0.58	664.68	646.40	682.96	13.99	13.61	14.38
Monaco	316.63	289.79	343.48	5.39	4.94	5.85	10.83	8.63	13.02	0.98	0.78	1.17	1859.09	1809.12	1909.06	39.14	38.09	40.19
Canada	114.42	100.62	128.21	1.95	1.71	2.18	2.29	1.44	3.15	0.21	0.13	0.28	663.60	635.76	691.44	13.97	13.38	14.56
France	20.78	18.42	23.14	0.35	0.31	0.39	0.45	0.30	0.61	0.04	0.03	0.05	118.71	114.03	123.40	2.50	2.40	2.60
Austria	150.19	135.33	165.04	2.56	2.31	2.81	6.12	4.80	7.44	0.55	0.43	0.67	876.73	847.81	905.65	18.46	17.85	19.07
Britain	327.62	301.80	353.44	5.58	5.14	6.02	15.34	12.87	17.82	1.38	1.16	1.61	1890.10	1843.66	1936.54	39.79	38.81	40.77
German	163.79	149.93	177.65	2.79	2.55	3.03	4.91	3.84	5.97	0.44	0.35	0.54	951.46	925.76	977.16	20.03	19.49	20.57
Hungarian	122.97	111.87	134.08	2.09	1.91	2.28	4.54	3.59	5.48	0.41	0.32	0.49	695.86	674.99	716.73	14.65	14.21	15.09
Belgium	316.44	290.82	342.07	5.39	4.95	5.83	10.64	8.55	12.72	0.96	0.77	1.15	1892.37	1845.32	1939.43	39.84	38.85	40.83
Italy	223.90	205.35	242.46	3.81	3.50	4.13	9.49	7.80	11.18	0.85	0.70	1.01	1304.29	1270.12	1338.45	27.46	26.74	28.18
Singapore	289.11	264.14	314.08	4.93	4.50	5.35	9.99	7.94	12.05	0.09	0.72	1.09	1665.71	1619.19	1712.24	35.07	34.09	36.05
Russia	83.06	75.22	90.91	1.42	1.28	1.55	4.17	3.40	4.95	0.38	0.31	0.45	472.72	457.76	487.68	9.95	9.64	10.27
Japan	72.06	62.14	81.98	1.23	1.06	1.40	4.27	3.21	5.32	0.38	0.29	0.48	401.55	381.53	421.57	8.45	8.03	8.88
USA	252.51	233.10	271.92	4.30	3.97	4.63	7.47	5.99	8.95	0.67	0.54	0.81	1451.44	1416.95	1485.92	30.56	29.83	31.28
Mexico	100.11	86.77	113.45	1.71	1.48	1.93	2.61	1.66	3.55	0.23	0.15	0.32	575.23	548.05	602.41	12.11	11.54	12.68
Brazil	114.59	99.69	129.49	1.95	1.70	2.21	1.50	0.75	2.25	0.14	0.07	0.20	652.52	622.36	682.67	13.74	13.10	14.37
Abu Dhabi	54.41	49.62	59.20	0.93	0.85	1.01	2.19	1.77	2.62	0.20	0.16	0.24	308.82	299.88	317.76	6.50	6.31	6.69

For peer review only

Quantifying alcohol audio-visual content in UK broadcasts of the 2018 Formula 1 Championship: a content analysis and population exposure.

Journal:	*BMJ Open*
Manuscript ID	bmjopen-2020-037035.R1
Article Type:	Original research
Date Submitted by the Author:	08-Apr-2020
Complete List of Authors:	Barker, Alexander; University of Nottingham, Division of Epidemiology and Public Health<br>Opazo-Breton, Magdalena; University of Nottingham, Division of Epidemiology and Public Health<br>Thomson, Emily; University of Nottingham<br>Britton, John; University of Nottingham, Division of Epidemiology<br>Grant-Braham, Bruce; Bournemouth University - Talbot Campus<br>Murray, Rachael; University of Nottingham, Division of Epidemiology and Public Health
**Primary Subject Heading**:	Public health
Secondary Subject Heading:	Public health
Keywords:	EPIDEMIOLOGY, PUBLIC HEALTH, STATISTICS & RESEARCH METHODS

SCHOLARONE™
Manuscripts

I, the Submitting Author has the right to grant and does grant on behalf of all authors of the Work (as defined in the below author licence), an exclusive licence and/or a non-exclusive licence for contributions from authors who are: i) UK Crown employees; ii) where BMJ has agreed a CC-BY licence shall apply, and/or iii) in accordance with the terms applicable for US Federal Government officers or employees acting as part of their official duties; on a worldwide, perpetual, irrevocable, royalty-free basis to BMJ Publishing Group Ltd ("BMJ") its licensees and where the relevant Journal is co-owned by BMJ to the co-owners of the Journal, to publish the Work in this journal and any other BMJ products and to exploit all rights, as set out in our licence.

The Submitting Author accepts and understands that any supply made under these terms is made by BMJ to the Submitting Author unless you are acting as an employee on behalf of your employer or a postgraduate student of an affiliated institution which is paying any applicable article publishing charge ("APC") for Open Access articles. Where the Submitting Author wishes to make the Work available on an Open Access basis (and intends to pay the relevant APC), the terms of reuse of such Open Access shall be governed by a Creative Commons licence – details of these licences and which Creative Commons licence will apply to this Work are set out in our licence referred to above.

Other than as permitted in any relevant BMJ Author's Self Archiving Policies, I confirm this Work has not been accepted for publication elsewhere, is not being considered for publication elsewhere and does not duplicate material already published. I confirm all authors consent to publication of this Work and authorise the granting of this licence.

For peer review only

Quantifying alcohol audio-visual content in UK broadcasts of the 2018 Formula 1 Championship: a content analysis and population exposure.

Alexander B. Barker[1], Magdalena Opazo Breton[1], Emily Thomson[1], John Britton[1], Bruce Grant-Braham[2], Rachael L. Murray[1]

[1]UK Centre for Tobacco and Alcohol Studies, Division of Epidemiology and Public Health, University of Nottingham, Clinical Sciences Building, City Hospital, Nottingham, NG5 1PB, United Kingdom

[2]University of Bournemouth, Dorset House, Talbot Campus, Fern Barrow, Poole, BH12 5BB, United Kingdom

Corresponding Author: Alexander B. Barker, alexander.barker@nottingham.ac.uk

Magdalena Opazo Breton, magdalena.opazo@nottingham.ac.uk

Emily Thomson, mzyet3@exmail.nottingham.ac.uk

John Britton, j.britton@outlook.com

Bruce Grant-Braham, BBraham@bournemouth.ac.uk

Rachael L. Murray, rachael.murray@nottingham.ac.uk

Word Count: 4084(excl. abstract and references)

Declaration of conflict of interests: Nothing to declare

Funding

This work was supported by the Medical Research Council [grant number MR/K023195/1] and the UK Centre for Tobacco and Alcohol Studies, with core funding from the British Heart Foundation, Cancer Research UK, Economic and Social Research Council and the Department of Health under the auspices of the UK Clinical Research Collaboration. The funders had no role in the study design, data collection and analysis, decision to publish or preparation of the manuscript.

For peer review only

For peer review only - http://bmjopen.bmj.com/site/about/guidelines.xhtml

Abstract

Objectives: Exposure to alcohol imagery is associated with subsequent alcohol use among young people, and UK broadcasting regulations protect young people from advertising and alcohol content in UK television. However, alcohol promotion during sporting events, a significant potential medium of advertising to children, is exempt. We report an analysis of and estimate the UK population exposure to, alcohol content, including branding, in UK broadcasts of the 2018 F1 Championship.

Setting: UK

Participants: None. Content analysis of broadcast footage of 21 2018 F1 Championship races on Channel 4, using 1-minute interval coding of any alcohol content, actual or implied use, other related content or branding. Census data and viewing figures were used to estimate gross and per capita alcohol impressions.

Results: Alcohol content occurred in all races, in 1613 (56%) one-minute intervals of race footage and 44 (13%) of intervals across 28% of advertisement breaks. The most prominent content was branding, occurring in 51% of race intervals and 7% of advertisement break intervals, appearing predominantly on billboard advertisements around the track, with the *Heineken* and *Johnnie Walker* brands being particularly prominent. The 21 races delivered an estimated 3.9 billion alcohol gross impressions (95% CI 3.6 – 4.3) to the UK population, including 154 million (95% CI 124 - 184) to children; and 3.6 billion alcohol gross impressions of alcohol branding, including 141 million impressions to children. Branding was also shown in race footage from countries where alcohol promotion is prohibited.

For peer review only - http://bmjopen.bmj.com/site/about/guidelines.xhtml

For peer review only

Conclusions: Alcohol content was highly prevalent in the 2018 F1 Championship broadcasts, delivering millions of alcohol impressions to young viewers. This exposure is likely to represent a significant driver of alcohol consumption among young people.

Article Summary

Strengths and limitations of the study

- This study builds on previous studies by estimating population exposure to alcohol content shown in F1 races
- Established methods were used to explore alcohol content in the 2018 F1 championship races
- This study only explored broadcasts where races occurred and not supplementary programmes
- Future studies should explore sales data to examine whether this exposure led to an increase in sales.

Introduction

Alcohol consumption in the UK is the 8th highest in Europe (1), and currently causes at least 7,000 UK deaths (2) and an estimated economic burden of between £21 and £52 billion each year (3). It is estimated that 1.6 million people in the UK have some form of alcohol dependence (4), alcohol misuse increases the risk of serious health conditions such as stroke, heart disease and cancer (5) Preventing alcohol morbidity and mortality is therefore a clear public health priority.

There is strong evidence that exposure to advertising or other alcohol audio visual content (AVC) in the media increases subsequent alcohol use by adolescents (6-9), and both commercial advertising of alcohol and alcohol content in broadcast programmes is

For peer review only

regulated in the UK to protect adolescents from exposure. However, Advertising Standards Authority commercial advertising regulatory codes do not cover broadcast footage of imagery arising from sponsorship of sporting events (10, 11); since this is programme content, it is regulated instead by the Office of Communications (Ofcom). The Ofcom Broadcasting Code (12) restricts depictions of alcohol misuse in programmes made for children, and the glamorisation of alcohol use in programmes broadcast before the 9 p.m. watershed (13) otherwise likely to be widely seen, heard or accessed by children without editorial justification (Section 1.10). The Code also restricts advertising within programmes, stating that 'no undue prominence may be given in programming to a product, service or trade mark' through the presence of or reference to a product where the is no editorial justification or the manner in which a product, service or trademark appears in programming (Section 9.5). However, Ofcom has no remit over sports sponsorship deals, such as a company sponsoring a stadium, a team or an individual sportsperson, and OfCom guidance states that the context of advertising is taken into account with a greater number of in situ advertising expected at sporting venues (14, 15). Alcohol advertising during televised sporting events is thus a potentially unregulated source of exposure to alcohol content for young people.

Whilst our previous research has shown that alcohol content and branding is highly prevalent in a sample of F1 Championship races (16), the total amount of content and branding shown through an entire annual championship, and the population exposure (particularly among young people) to this content remains unknown. In 2018, Heineken continued to sponsor the F1 Championship races (17) which were viewed by 45.5 million

For peer review only - http://bmjopen.bmj.com/site/about/guidelines.xhtml

viewers in the UK (18), an estimated 14% of which were aged under 25 (19). We have therefore quantified alcohol AVC in all 21 races from the 2018 F1 Championship and estimated UK population exposure, including child exposure, to this content. We have also compared the amount of alcohol imagery in the 2018 race series with our earlier analysis of footage from the 2017 competition.

Methods

The 2018 Formula 1 Championship took place between 15th March 2018 and 1st December 2018 and featured races in twenty-one countries. Ten of these races were broadcast in the UK live and in full by Channel 4, and eleven in selected highlight form. These broadcasts were recorded in their entirety (start of the programme until the end of the credits), viewed and coded using the one-minute interval period method previously described (16, 20-23), coding the presence of audio-visual alcohol content every one-minute interval in the following categories:

Actual Use: Use of alcohol onscreen by any person (for example, seeing a person actually consume alcohol on screen

Implied Use: Any implied alcohol use without any actual use on screen (for example, seeing a person holding a drink/bottle of alcohol, but not actually consuming alcohol).

Other Alcohol Reference: The presence onscreen of alcohol or other related materials (for example bottles or beer pumps not currently in use, or advertising materials)

Brand Appearance: The presence of clear and unambiguous branding (for example, when a brand is identified on screen). Brand appearances were divided into the following

For peer review only - http://bmjopen.bmj.com/site/about/guidelines.xhtml

categories; Alcoholic and 0% or low alcohol products, products which, while not an alcohol product, share branding with alcoholic products.

Any Alcohol Content: Any of the above.

One-minute intervals were used rather than shorter intervals used in other studies (24), due to the length of the programme being coded and for practical issues around the amount of time required to code in 10 second intervals. For coding purposes, multiple instances of the same category in the same one-minute period were considered to be single event, whilst instances that ran into consecutive one-minute periods were coded as separate events. Instances in different categories in the same interval were recorded as different events. Alcohol content was recorded in two categories in the same interval if the content met the criteria for two categories, for example if a driver drank from a branded bottle, this would be recorded as actual use and a brand appearance. Ten percent of the coding was checked by a second author (ET) to ensure accuracy and reliability in the coding method.

We estimated UK audience exposure using viewing data from *Digital.I* (25) and UK mid-year population estimates for 2017 (26) combined with numbers of alcohol appearances (every minute containing alcohol use, implied use, other references or branding) to estimate gross (the total number of impressions delivered to the UK population) and per capita (the number of impressions delivered to each person) one-minute impressions by age group, using previously reported methods (27-29). The method involves combining viewership (obtained from viewing figures) with the number of intervals containing alcohol appearances per episode to calculate gross impressions as the estimated number of exposures delivered. Dividing gross impressions by population mid-year estimates provided per capita impressions, the estimated number of alcohol impressions delivered to each

For peer review only - http://bmjopen.bmj.com/site/about/guidelines.xhtml

For peer review only

person. Both gross and per capita impressions were computed by age group. Analyses were conducted in IBM SPSS Statistics (V.24) and Microsoft Excel (2013). The confidence level was set to 95%. We compared content with findings of our earlier studies alcohol content in a sample of F1 races from 2017 (16) using chi-square analysis.

Patient and public involvement

No patients were involved in this study

Results

We coded a total of 3396 one-minute intervals, of which 2899 were from race footage, and 497 from advertisement breaks, all of which were broadcast by Channel 4 on a Sunday, with start times ranging from 06:00 to 23:00 and a mean broadcast duration (including advertisement breaks) of 2 hours 21 minutes (range = 1 hour 34 minutes to 3 hours 7 minutes). Inter-rater coding agreement was 98.98% of 1-minute intervals in the sample double coded, with differences resolved upon discussion and multiple viewings.

Any Alcohol Content

In total, 1613 (56%) one-minute intervals of racing, and 44 (13%) one-minute advertisement intervals contained any alcohol content, with occurrences in all twenty-one races and 28% of advertisement breaks (Table 1). The majority of alcohol content occurred during race footage.

Table 1: Alcohol AVC content by type and location

For peer review only

Type of content	During race broadcast N (%)	During advertisements N (%)
Any Alcohol Content	1613 (56)	44 (9)
Actual Use	27 (1)	2 (2)
Implied use	92 (3)	34 (7)
Other Alcohol References	1571 (54)	38 (8)
Brand Appearance	1490 (51)	37 (7)
Heineken branding	1029 (35)	2 (2)

Actual Use

Alcohol consumption during race footage was limited to consumption of a sparkling drink on the winner's podium, identified from branding on the bottle or podium as Carbon Champagne, which occurred at the end of eighteen races (86%) in a total of 25 (1%) one-minute intervals. There were ten advertisement breaks depicting alcohol consumption, across 11 one-minute intervals (<1%).

Implied Use

Implied alcohol use was observed in 92 (3%) race intervals, predominantly the sparkling drink being sprayed from the winner's podium. Implied use was seen in 34 one-minute intervals during advertisement breaks, and predominantly comprised imagery of people holding drinks during advertisements for alcohol brands.

Other Alcohol References

Alcohol references, predominantly involving billboard and car brand advertising, occurred in 1571 race intervals (46% of all race intervals), including 'When you drink, never drive' billboards, which appeared in 241 (7%) of race intervals. Alcohol references also occurred in

For peer review only

For peer review only - http://bmjopen.bmj.com/site/about/guidelines.xhtml

38 (8%) one-minute intervals from advertisement breaks, mostly consisting of bottles or branded items (such as beer pumps) (Table 1).

Brand Appearances

Alcohol product branding in billboard advertisements or on the side of cars or racing suits, occurred in 1490 (51%) race intervals across all 21 races. The most common brands observed were *Heineken,* which occurred in 1031 intervals and appeared 8795 times; and *Johnnie Walker,* which appeared in 608 intervals and appeared on screen 4364 times. *Heineken 0.0,* a non-alcoholic lager which shared the *Heineken* brand name and imagery, occurred in 120 one-minute intervals (8% of the 1490 intervals containing branding) and appeared 548 times. Alcohol branding occurred in 37 (7%) advertisement break intervals, with the most common brand being *Heineken* (Table 1). A list of all the brands observed is presented in Figure 1.

Figure 1: Brands identified in race footage and advertisements

Figure 1 here

In addition to billboard advertising, computer-generated advertising added to highlights programmes were seen in 17 intervals during two races (Canada and Austria). These intervals featured a red star, reminiscent of the *Heineken* logo, superimposed over the track.

For peer review only - http://bmjopen.bmj.com/site/about/guidelines.xhtml

Differences between race venues

The amount of alcohol content and alcohol branding was highest in Monaco GP and lowest in the Bahrain GP (table 2)

For peer review only - http://bmjopen.bmj.com/site/about/guidelines.xhtml

Table 2: Comparison of alcohol content between race venues (in order of broadcast)

	Number of 1-minute intervals (% proportion of race intervals)																				
	**Australia**	**Bahrain**	**China**	**Azerbaijan**	**Spain**	**Monaco**	**Canada**	**France**	**Austria**	**Britain**	**Germany**	**Hungary**	**Belgium**	**Italy**	**Singapore**	**Russia**	**Japan**	**USA**	**Mexico**	**Brazil**	**Abu Dhabi**
Any Alcohol Content	79 (55%)	18 (12%)	92 (73%)	81 (43%)	48 (45%)	129 (80%)	90 (96%)	15 (15%)	82 (49%)	110 (66%)	65 (55%)	55 (48%)	117 (74%)	88 (76%)	122 (71%)	40 (34%)	75 (50%)	84 (55%)	97 (83%)	102 (87%)	24 (15%)
Alcohol Use	1 (1%)	0 (0%)	1 (1%)	1 (1%)	2 (2%)	3 (2%)	1 (1%)	2 (2%)	2 (1%)	1 (1%)	1 (1%)	1 (1%)	2 (1%)	1 (1%)	1 (1%)	2 (2%)	1 (1%)	1 (1%)	1 (1%)	1 (1%)	1 (1%)
Implied Use	5 (1%)	1 (1%)	2 (2%)	4 (2%)	7 (7%)	5 (3%)	5 (5%)	4 (4%)	2 (1%)	2 (1%)	4 (3%)	5 (4%)	2 (1%)	6 (5%)	0 (0%)	5 (4%)	1 (1%)	5 (3%)	13 (11%)	10 (9%)	4 (2%)
Other Alcohol References	76 (53%)	13 (9%)	92 (73%)	77 (41%)	43 (41%)	126 (78%)	90 (96%)	15 (15%)	80 (48%)	109 (66%)	63 (53%)	53 (46%)	117 (74%)	87 (75%)	121 (70%)	38 (33%)	75 (50%)	82 (54%)	91 (78%)	102 (87%)	21 (13%)
Brand Appearance	59 (41%)	10 (7%)	92 (73%)	68 (36%)	39 (37%)	121 (75%)	84 (89%)	11 (11%)	79 (47%)	108 (66%)	62 (53%)	48 (42%)	114 (72%)	87 (75%)	121 (70%)	36 (31%)	63 (42%)	82 (54%)	89 (76%)	101 (86%)	16 (10%)
Any Alcohol Content	79 (55%)	18 (12%)	92 (73%)	81 (43%)	48 (45%)	129 (80%)	90 (96%)	15 (15%)	82 (49%)	110 (66%)	65 (55%)	55 (48%)	117 (74%)	88 (76%)	122 (71%)	40 (34%)	75 (50%)	84 (55%)	97 (83%)	102 (87%)	24 (15%)
Alcohol Use	1 (1%)	0 (0%)	1 (1%)	1 (1%)	2 (2%)	3 (2%)	1 (1%)	2 (2%)	2 (1%)	1 (1%)	1 (1%)	1 (1%)	2 (1%)	1 (1%)	1 (1%)	2 (2%)	1 (1%)	1 (1%)	1 (1%)	1 (1%)	1 (1%)
Implied Use	5 (1%)	1 (1%)	2 (2%)	4 (2%)	7 (7%)	5 (3%)	5 (5%)	4 (4%)	2 (1%)	2 (1%)	4 (3%)	5 (4%)	2 (1%)	6 (5%)	0 (0%)	5 (4%)	1 (1%)	5 (3%)	13 (11%)	10 (9%)	4 (2%)
Other Alcohol References	76 (53%)	13 (9%)	92 (73%)	77 (41%)	43 (41%)	126 (78%)	90 (96%)	15 (15%)	80 (48%)	109 (66%)	63 (53%)	53 (46%)	117 (74%)	87 (75%)	121 (70%)	38 (33%)	75 (50%)	82 (54%)	91 (78%)	102 (87%)	21 (13%)
Brand Appearance	59 (41%)	10 (7%)	92 (73%)	68 (36%)	39 (37%)	121 (75%)	84 (89%)	11 (11%)	79 (47%)	108 (66%)	62 (53%)	48 (42%)	114 (72%)	87 (75%)	121 (70%)	36 (31%)	63 (42%)	82 (54%)	89 (76%)	101 (86%)	16 (10%)
Heineken	33 (23%)	0 (0%)	85 (67%)	59 (32%)	0 (0%)	114 (70%)	82 (87%)	1 (1%)	69 (41%)	82 (49%)	14 (12%)	40 (35%)	44 (28%)	74 (64%)	86 (50%)	1 (1%)	65 (43%)	12 (8%)	71 (60%)	96 (82%)	1 (1%)
Heineken 0.0	0 (0%)	0 (0%)	0 (0%)	0 (0%)	30 (28%)	0 (0%)	0 (0%)	0 (0%)	0 (0%)	0 (0%)	0 (0%)	0 (0%)	0 (0%)	36 (31%)	0 (0%)	31 (26%)	0 (0%)	0 (0%)	0 (0%)	0 (0%)	0 (0%)

For peer review only

UK Population Exposure

We estimate that the 21 race broadcasts delivered 3.9 billion alcohol gross impressions (95% CI 3.6 – 4.3) to the UK population, including 154 million (95% CI 124 – 184) to children aged under 16. Alcohol impressions per capita were highest (average 513.11 (95%CI 499.96 – 526.25)) in the 55-64 age group and were higher in men than women (513.11 (95% CI 499.96 – 526.25) and (433.50 (95%CI 420.98 – 446.02) respectively). Children received an average 12.25 (95% CI 9.84 – 14.66) per capita impressions. Of these impressions, 3.6 billion (95% CI 3.3 – 4.0) were of alcohol branding and including 141 million (95% CI 113 – 169) to children. (See supplementary table 1 for a breakdown of gross and per capita impressions per race). We estimate that the computer-generated branding features in 2 races delivered 269.34 million gross impressions to the UK population including 1.69 million to children.

Comparison to 2017 Championship

Comparison with our earlier analysis of alcohol content in the 2017 F1 championship (16) demonstrated significantly greater alcohol content in the 2018 season, particularly in relation to the proportion of intervals including alcohol branding, and especially Heineken advertising (Table 3). Heineken 0.0 advertising was significantly less prevalent in the 2018 footage (Table 3). In our previous content analysis (16), Heineken 0.0 branding was seen at only 2 events, the Spanish Grand Prix and the Monaco Grand Prix. In the 2018 Monaco Grand Prix, billboard advertising for Heineken 0.0 had been replaced with conventional Heineken imagery.

Table 3: Proportion (%) of race footage containing alcohol content in the 2018 and 2017 Championships.

	2018 Championship	2017 Championship	p-value
**Alcohol**			
Any Alcohol Content	56%	46%	<0.01
Actual Alcohol Use	1%	1%	1
Implied Alcohol Use	3%	4%	0.1498
Other Alcohol reference	54%	44%	<0.01
Alcohol Branding	51%	35%	<0.01
Heineken	35%	13%	<0.01

Heineken 0.0	3%	14%	<0.01

Discussion

This study demonstrates that audio-visual alcohol content, including branding and particularly *Heineken* and *Johnnie Walker* branding, was highly prevalent in broadcast footage of the 2018 F1 championship races, and that the overall amount of alcohol content in 2018 was greater than in 2017 (16), largely because of substitution of zero-alcohol with alcoholic *Heineken* branding. The alcohol content in the broadcast race footage generates substantial population exposure, delivering billions of viewer impressions. These included more than 100 million gross branded alcohol impressions to children aged under 16.

Our study thus provides evidence that UK broadcast footage of the F1 Championship is a significant source of exposure to alcohol content and advertising for children. Alcohol content was prevalent in all races, even races which took place in countries with alcohol advertising restrictions. Alcohol advertising, through sponsorship deals, during these events appears to be changing with the inclusion of computer-generated alcohol advertising, which was introduced in the 2018 Championship. Since the F1 Championships are international events with global audiences, the UK population exposure figures likely represent a very small proportion of the true total global exposure.

Some alcohol producers associate their advertising with responsible drinking messages. Heineken's 5-year F1 sponsorship has been heavily criticised *for "*linking a popular motor sport to a significant cause of avoidable physical, mental and social harm and more specifically one of the major killers on our roads, drink driving"(30). This global campaign incorporates Heineken's characteristic red star and green branding on billboards which also

For peer review only - http://bmjopen.bmj.com/site/about/guidelines.xhtml

have a prominent *"When You Drive, Never Drink"* message (31). Indeed Heineken has said that it will use F1 to promote this campaign (32) and the *Heineken 0.0* brand (33). We have previously argued that *Heineken 0.0* advertising may be acting as an alibi for alcohol products (16), not least because they share the same brand imagery (a star in the case of Heineken) on both alcohol and low alcohol products, potential consumers, and particularly young people, may well not distinguish advertising for alcoholic and non-alcoholic *Heineken* products. This industry tactic could potentially be serving a subtle public relations function, engendering goodwill with potential consumers of the brand while increasing their promotion of their alcohol products, promoting brand preference and product consumption (34, 35). The current study has identified that the proportion of *Heineken 0.0* advertising has decreased significantly since our 2017, with billboards for *Heineken 0.0* from 2017 being replaced with regular *Heineken* billboards.

The majority of the F1 Championship races (18 of 21) were broadcast on a Sunday before the 9 p.m. watershed, a time when children are likely to be watching what their parents are watching (36). Channel 4 state that they '*abide*' by the Office of Communications (Ofcom) Broadcasting Code (12), which considers factors that determine whether a programme should be shown including *'the likely number and age range of children watching, taking into account school time, weekends and holidays'(37)*. The current study has demonstrated that the broadcast footage of the F1 Championship delivers millions of branded alcohol content impressions to children. Furthermore, the Ofcom code also states that *'Before the watershed, …the misuse of alcohol must never be condoned, encouraged or glamorised and scenes showing such material should generally be avoided unless there is editorial justification'* (12). All of the F1 races featured scenes of drivers celebrating their win by

drinking from and spraying bottles of champagne, a traditional celebration in F1 (38), which could potentially be associating driving with alcohol use (39) and glamourising alcohol use by showing drivers consuming alcohol in what F1's CEO considers to be a glamourous sport (40).

Rule 9.5 of the Ofcom Broadcasting Code (12) states that 'no undue prominence may be given in programming to a product, service or trade mark' through the presence of or reference to a product where the is no editorial justification or the manner in which a product, service or trademark appears in programming. Whilst this rule takes into account the context and in-situ advertising at venues is editorially justified, the current study found that computer generated Heineken branding was added to F1 race highlights programmes, through the inclusion of a red star superimposed over the track similar to Heineken's red star logo representing an apparent clear breach of the Ofcom broadcasting code. Channel 4 was recently found to be in breach of the code for their Formula 1. We reported this to Ofcom (15) which noted that the two Grands Prix containing computer-generated alcohol advertising were broadcast before an Ofcom ruling which found Channel 4 to be in breach of the code for their F1 coverage featuring computer-generated 'Rolex' branding (a 'Rolex' clock face superimposed over footage of The Singapore Flyer, a large Ferris wheel) (41), and expect broadcasters to take the precedent of the 'Rolex' ruling into account for subsequent broadcasts.

Whilst there is scope under Ofcom's powers to regulate content in programmes broadcast in the UK, Ofcom has no remit over sports sponsorship deals, and these are therefore a potentially unregulated source of exposure to alcohol content for young people. Furthermore, the F1 Championship races are broadcast internationally, which complicates regulating this content. The

UK population exposure to alcohol audio-visual content in the 2018 Formula 1 Championship.

For peer review only - http://bmjopen.bmj.com/site/about/guidelines.xhtml

Framework Convention for Tobacco Control (42) is a treaty negotiated under the auspices of the World Health Organisation which provides a regulatory strategy to address addictive substances, has placed an international, comprehensive ban on the advertising, promotion and sponsorship of tobacco products (43). A similar comprehensive ban on the advertising, promotion and sponsorship of alcohol products would prevent young people being exposed to this currently unregulated alcohol promotion.

Alcohol content and brand prevalence differed between race venues, possibly due to differences in national restrictions on alcohol advertising (44). The races with the least alcohol content were Bahrain, Abu Dhabi and France; Bahrain and Abu Dhabi are events in Islamic countries were alcohol advertising is prohibited, while France has statutory legislation, 'the Loi Evin'(45), prohibiting alcohol marketing and advertising by placing a total ban on the direct or indirect advertising of all alcoholic beverages over 1.2% ABV on television and prohibiting sponsorship of sports events by alcohol companies (46). Alcohol advertising did occur in UK broadcast footage of races in countries where alcohol advertising is prohibited, such as Russia, France, and Bahrain, raising concerns that alcohol brands are bypassing country-specific regulations in the F1 races. Race footage featuring billboard advertisements are also broadcast worldwide, which could circumvent alcohol advertising regulations in a similar way to the UK's lack of regulation covering advertisements at sporting venues (10, 11, 14, 15).

Our study is limited to UK broadcasts shown on Channel 4, it is possible that other broadcasts, such as those shown on Sky one, showed different footage. The current quantification and population exposure only explored UK footage, and therefore is unable to estimate the amount or exposure to content broadcast in other countries, however, it is

For peer review only - http://bmjopen.bmj.com/site/about/guidelines.xhtml

likely that our findings apply much more widely than in the UK. Channel 4 also broadcast qualifying race and pre and post-event programmes, in this content analysis we focused on the broadcast containing the actual race as specified by Channel 4, because of this it is likely that this content analysis underestimates the amount of alcohol AVC shown in the F1 Championship.

Whilst the current study found that alcohol branding was highly prevalent in broadcast footage of the 2018 F1 championship races, we currently do not know whether this led to increased alcohol sales. Future studies should explore sales data for prevalent brands to identify if the presence of branding in such events led to an increase in sales of branded goods featured in the Championship races. Channel 4 also broadcast other F1 programmes, including qualifying race and pre and post-event programmes, in this content analysis we focused on the broadcast containing the actual race as specified by Channel 4, rather than qualifying or post event programmes. It is therefore likely that this content analysis underestimates the amount of alcohol AVC shown in the F1 Championship as a whole, and the population exposure to this content.

Conclusion

Alcohol content shown on TV, including alcohol advertisements and branding, is known to have an effect on the uptake of alcohol use in young people (47). Alcohol content broadcast during the F1 races resulted in millions of gross alcohol content impressions being delivered to children, and this exposure is likely to lead to subsequent alcohol use. We also found computer-generated branding in the Formula 1 races broadcast in the UK, contravening an

For peer review only

For peer review only - http://bmjopen.bmj.com/site/about/guidelines.xhtml

earlier Ofcom ruling, and alcohol advertising in countries where this is prohibited. Restrictions on and enforcement of alcohol AVC during sporting events are needed to protect children and adolescents from this avenue of alcohol advertising.

Declarations

Patient consent for publication: Not required.

Competing interests: The authors declare that they have no competing interests.

Funding: This work was supported by the Medical Research Council [grant number MR/K023195/1] and the UK Centre for Tobacco and Alcohol Studies, with core funding from the British Heart Foundation, Cancer Research UK, Economic and Social Research Council and the Department of Health under the auspices of the UK Clinical Research Collaboration. The funders had no role in the study design, data collection and analysis, decision to publish or preparation of the manuscript.

Author's contributions: ABB led the data coding and data analysis and contributed to the manuscript. ET double coded the data to ensure reliability. MOP supported data analysis. BG contributed with interpreting the findings of the study. JB and RM contributed to drafting the manuscript. All authors read and approved the final manuscript.

Acknowledgements: Not applicable

Availability of data and materials: Data and materials are available from the lead author upon reasonable request.

For peer review only - http://bmjopen.bmj.com/site/about/guidelines.xhtml

References

1. United European Gastroenterology. Alcohol and Digestive Cancers 2017 [Available from: https://www.ueg.eu/publications/alcohol-and-digestive-cancers-report/.
2. Office on National Statistics. Statistics on Alcohol; England 2017. http://www.content.digital.nhs.uk/catalogue/PUB23940/alc-eng-2017-rep.pdf: NHS England; 2017.
3. Public Health England. The public health burden of alcohol and the effectiveness and cost-effectiveness of alcohol control policies: an evidence review. 2016.
4. Public Health E. Alcohol treatment in England 2013-14. 2014.
5. NHS. Alcohol misuse 2020 [Available from: https://www.nhs.uk/conditions/alcohol-misuse/.
6. Anderson P, De Brujin A, Angus K, Gordon R, Hastings G. Impact of alcohol advertising and media exposure on adolescent alcohol use: a systematic review of longitudinal studies. Alcohol and Alcoholism. 2009;44(3):229-43.
7. Smith L, Foxcroft DR. The effects of alcohol advertising, marketing and portrayal on drinkin behaviour in young people: systematic review of prospective cohort studies. BMC Public Health. 2009;9(51):1-11.
8. Hanewinkel R, Sargent JD, Hunt K, Sweeting H, Engels RC, Scholte RH. Portrayal of alcohol consumption in movies and drinking initiation in low-risk adolescents. Pediatrics. 2014;133:973-82.
9. Chang F, Miao N, Lee C, Chiu C, Lee S. The association of media exposure and media literacy with adolescent alcohol and tobacco use. Journal of Health Psychology. 2016;21(4):513-25.
10. Advertising Standards Authority. The CAP code 2019 [Available from: https://www.asa.org.uk/type/non_broadcast/code_folder/scope-of-the-code.html.
11. Advertising Standards Authority. The Broadcast Code 2019 [Available from: https://www.asa.org.uk/codes-and-rulings/advertising-codes/broadcast-code.html.
12. Ofcom. The Ofcom Broadcasting Code (with the Cross-promotion Code and the On Demand Programme Service Rules) 2017 [Available from: https://www.ofcom.org.uk/tv-radio-and-on-demand/broadcast-codes/broadcast-code.
13. Ofcom. What is the Watershed? 2013 [Available from: https://www.ofcom.org.uk/tv-radio-and-on-demand/advice-for-consumers/television/what-is-the-watershed.
14. Ofcom. Guidance Notes: Section Nine: Commercial references in television programming 2016 [Available from: https://www.ofcom.org.uk/__data/assets/pdf_file/0014/33611/section9_may16.pdf.
15. Ingram P. Letter regarding Heineken branding in Channel four's coverage of Formula 1, Ref.: 00626184 2018 [Available from: https://ukctas.net/pdfs/20181115-ofcom-letter-to-Alex-Barker.pdf.
16. Barker AB, Britton J, Grant-Braham B, Murray RL. Alcohol Audio-Visual Content in Formula 1 television broadcasting. BMC Public Health. 2018;18(1155).
17. Reuters. Heineken and F1 announce multi-year deal 2016 [Available from: https://www.reuters.com/article/us-motor-f1-canada-heineken-idUSKCN0YV2A4.
18. Sports Pro Media. Report: F1 sees UK TV audience fall by 8.6m in 2019 2020 [Available from: https://www.sportspromedia.com/news/f1-uk-tv-audience-fall-2019-sky-sports-channel-4.
19. Forbes. F1 reveals that just 14% of its viewers are under 25 2019 [Available from: https://www.forbes.com/sites/csylt/2019/01/13/f1-reveals-that-just-14-of-its-viewers-are-under-25/#2f9cd11f6d5c.
20. Lyons A, McNeill A, Britton J. Tobacco imagery on prime time UK television. Tobacco Control. 2013;23(3):257-63.
21. Barker AB, Whittamore KH, Britton J, Murray RL, Cranwell J. Audio-visual alcohol content in UK television. Journal of Public Health. 2018;Online First.
22. Barker AB, Smith J, Hunter A, Britton J, Murray RL. Tobacco and alcohol audio-visual content in Netflix and Amazon Prime Instant Video original programming. BMJ Open. 2019.

For peer review only

For peer review only - http://bmjopen.bmj.com/site/about/guidelines.xhtml

23. Barker AB, Whittamore KH, Britton J, Cranwell J. A content analysis of tobacco content in UK television. Tobacco Control. 2018;Online First.
24. Cranwell J, Murray R, Lewis S, Leonardi-Bee J, Dockrell M, Britton J. Adolescents' exposure to tobacco and alcohol content in YouTube music videos. Addiction. 2015;110(4):703-11.
25. Digital I. Digital.I: A fresh perspective on TV viewing figures 2018 [Available from: http://www.digital-i.com.
26. Office for National Statistics. Population estimates for the UK, England and Wales, Scotland and Northern Ireland: Mid 2017 2018 [Available from: https://www.ons.gov.uk/peoplepopulationandcommunity/populationandmigration/populationestimates/bulletins/annualmidyearpopulationestimates/mid2017.
27. Cranwell J, Opazo-Breton M, Britton J. Adult and adolescent exposure to tobacco and alcohol content in contemporary YouTube music videos in Great Britain: a population estimate. J Epidemiol Community Health. 2016;70(5):488-92.
28. Barker AB, Opazo Breton M, Cranwell J, Britton J, Murray RL. Population exposure to smoking and tobacco branding in the UK reality show ‘Love Island’. Tobacco Control. 2018.
29. Sargent JD, Tanski SE, Gibson J. Exposure to movie smoking among US adolescents aged 10 to 14 years: a population estimate. Pediatrics. 2007;119(5):e1167-76.
30. Crash. Heineken F1 deal leads to fresh calls for alcohol sponsorship ban 2016 [Available from: https://www.crash.net/f1/news/231262/1/heineken-f1-deal-leads-to-fresh-calls-for-alcohol-sponsorship-ban.
31. Eurocare. Formula 1 puts Heineken in the driving seat 2016 [Available from: http://www.eurocare.org/media_centre/press_releases/formula_1_puts_heineken_in_the_driving_seat.
32. Heineken. When You Drive, Never Drink. 2015;2018.
33. Heineken. Heineken 0.0: Great taste, all natural, only 69 calories and zero alcohol 2017 [Available from: https://www.theheinekencompany.com/media/media-releases/press-releases/2017/05/2104120.
34. Smith SW, Atkin CK, Roznowski J. Are 'Drink Responsibly' alcohol campaigns strategically ambiguous? Health Communication. 2006;20(1):1-11.
35. Jones SC, Hall S, Kypri K. Should I drink responsibly, safely or properly? Confusing messages about reducing alcohol related harm. PLOS One. 2017;12(9):e0184705.
36. Jago R, Thompson J, Sebire S, Wood L, Pool L, Zahra J, et al. Cross-sectional associations between the screen-time of parents and young children: differences by parent and child gender and day of the week. International Journal of Behavioral Nutrition and Physical Activity. 2014;11(54):1-8.
37. Channel 4. Producers Handbook 2017 [Available from: http://www.channel4.com/producers-handbook/.
38. Times T. Obituary: Dan Gurney 2018 [Available from: https://www.thetimes.co.uk/article/obituary-dan-gurney-c689k76k6.
39. Eurocare. Alcohol advertising and sponsorship in Formula One: A Dangerous Cocktail 2015 [Available from: http://www.ias.org.uk/uploads/pdf/IAS%20reports/ALCOHOL_ADVERTISING_AND_SPONSORSHIP_IN_FORMULA_ONE__A_DANGEROUS_COCKTAIL_MAY_2015.pdf.
40. Tass: Russian News Agency. Formula One racing to remain 'glamorous sport' even without grid girls, vows F1 CEO Carey [Available from: https://tass.com/sport/989870.
41. Ofcom. Ofcom broadcast and on demand bulletin 2018 [Page 41]. Available from: https://www.ofcom.org.uk/__data/assets/pdf_file/0027/117468/broadcast-on-demand-bulletin-issue-359.pdf.
42. World Health Organization. Framework Convention on Tobacco Control 2005 [Available from: https://www.who.int/fctc/text_download/en/.

For peer review only - http://bmjopen.bmj.com/site/about/guidelines.xhtml

43. World Health Organisation. Guidelines for the implementation of Article 13 of the WHO Framework Convention on Tobacco Control 2005 [Available from: https://www.who.int/fctc/guidelines/article_13.pdf.
44. World Health Organisation. Management of substance abuse: country profiles 2014 2014 [Available from: http://www.who.int/substance_abuse/publications/global_alcohol_report/profiles/en/.
45. Court of Justice of the European Communities: Opinion of First Advocate General in Cases C-262/02 and C-429/02, (2004).
46. Institute of Alcohol Studies. Alcohol advertising in the European Union 2019 [Available from: http://www.ias.org.uk/Alcohol-knowledge-centre/Marketing/Factsheets/Alcohol-advertising-in-the-European-Union.aspx.
47. Morgenstern M, Isansee B, Sargent JD, Hanewinkel R. Exposure to alcohol advertising and teen drinking. Preventive Medicine. 2011;2(1):146-51.

For peer review only

For peer review only - http://bmjopen.bmj.com/site/about/guidelines.xhtml

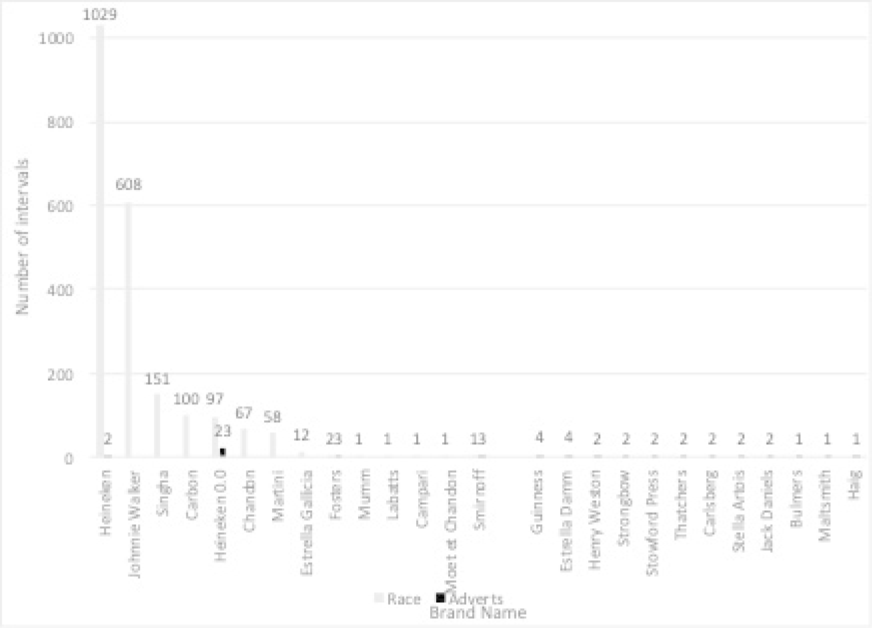

Figure 1: Brands identified in race footage and advertisements

38x30mm (300 x 300 DPI)

Supplementary table 1: Gross and per capita impressions per race by age

	Total						Child <16						Adult >16					
**Race**	**Gross impressions (Million)**	**95% Confidence Interval**		**Per Capita Impressions**	**95% Confidence Interval**		**Gross impressions (Million)**	**95% Confidence Interval**		**Per Capita Impressions**	**95% Confidence Interval**		**Gross impressions (Million)**	**95% Confidence Interval**		**Per Capita Impressions**	**95% Confidence Interval**	
Australia	170.91	153.96	187.87	2.57	2.32	2.83	8.47	6.81	10.14	0.67	0.54	0.80	995.23	962.19	1028.26	18.50	17.88	19.11
Bahrain	61.65	65.86	66.45	0.93	0.86	1.00	3.70	3.17	4.22	0.29	0.25	0.33	353.73	345.15	362.31	6.57	6.42	6.73
China	213.08	192.98	233.19	3.21	2.91	3.51	8.54	6.79	10.32	0.68	0.54	0.82	1264.72	1225.82	1303.63	23.51	22.78	24.23
Azerbaijan	268.28	247.49	289.07	4.04	3.73	4.35	17.55	15.20	19.91	1.39	1.21	1.58	1528.01	1490.98	1565.04	28.40	27.71	29.09
Spain	132.58	121.36	143.81	2.00	1.83	2.17	6.21	5.13	7.28	0.49	0.41	0.58	752.84	732.14	773.54	13.99	13.61	14.38
Monaco	358.17	327.80	388.53	5.39	4.94	5.85	12.29	9.80	14.78	0.98	0.78	1.17	2105.66	2049.07	2162.26	39.14	38.09	40.19
Canada	129.43	113.82	145.03	1.95	1.71	2.18	2.60	1.63	3.58	0.21	0.13	0.28	751.62	720.08	783.15	13.97	13.38	14.56
France	23.50	20.84	26.17	0.35	0.31	0.39	0.51	0.34	0.69	0.04	0.03	0.05	134.46	129.15	139.76	2.50	2.40	2.60
Austria	169.89	153.08	186.69	2.56	2.31	2.81	6.94	5.45	8.44	0.55	0.43	0.67	993.01	960.25	1025.76	18.46	17.85	19.07
Britain	370.60	341.39	399.80	5.58	5.14	6.02	17.42	14.61	20.23	1.38	1.16	1.61	2140.79	2088.19	2193.39	39.79	38.81	40.77
German	185.28	169.60	200.96	2.79	2.55	3.03	5.57	4.36	6.77	0.44	0.35	0.54	1077.65	1048.54	1106.76	20.03	19.49	20.57
Hungarian	139.10	126.54	151.66	2.09	1.91	2.28	5.15	4.08	6.22	0.41	0.32	0.49	788.15	764.51	811.79	14.65	14.21	15.09
Belgium	357.95	328.96	386.94	5.39	4.95	5.83	12.07	9.71	14.44	0.96	0.77	1.15	2143.36	2090.07	2196.66	39.84	38.85	40.83
Italy	253.27	232.28	274.26	3.81	3.50	4.13	10.77	8.85	12.69	0.85	0.70	1.01	1477.27	1438.58	1515.97	27.46	26.74	28.18
Singapore	327.03	298.79	355.28	4.93	4.50	5.35	11.34	9.01	13.67	0.09	0.72	1.09	1886.64	1833.94	1939.34	35.07	34.09	36.05
Russia	93.96	85.08	102.84	1.42	1.28	1.55	4.73	3.86	5.61	0.38	0.31	0.45	535.42	518.47	552.37	9.95	9.64	10.27
Japan	81.51	70.29	92.74	1.23	1.06	1.40	4.84	3.64	6.04	0.38	0.29	0.48	454.81	432.13	477.48	8.45	8.03	8.88
USA	285.63	263.68	307.59	4.30	3.97	4.63	8.48	6.80	10.16	0.67	0.54	0.81	1642.94	1604.88	1683.01	30.56	29.83	31.28
Mexico	113.24	98.16	128.33	1.71	1.48	1.93	2.96	1.89	4.03	0.23	0.15	0.32	651.52	620.74	682.31	12.11	11.54	12.68
Brazil	129.62	112.77	146.47	1.95	1.70	2.21	1.70	0.85	2.55	0.14	0.07	0.20	739.06	704.91	773.21	13.74	13.10	14.37
Abu Dhabi	61.55	56.13	66.97	0.93	0.85	1.01	2.49	2.01	2.97	0.20	0.16	0.24	349.78	339.66	359.90	6.50	6.31	6.69

For peer review only
